# Ferritin Level Is Positively Associated with Chronic Kidney Disease in Korean Men, Based on the 2010–2012 Korean National Health and Nutrition Examination Survey

**DOI:** 10.3390/ijerph13111058

**Published:** 2016-10-29

**Authors:** Hee-Taik Kang, John A. Linton, Soon Kil Kwon, Byoung-Jin Park, Jong Hun Lee

**Affiliations:** 1 Department of Family Medicine, College of Medicine, Chungbuk National University, Chungbuk 28644, Korea; kanght0818@gmail.com; 2 Department of Family Medicine, Severance Hospital, Yonsei University, Seoul 03722, Korea; yohan@yuhs.ac; 3 Division of Nephrology, Department of Internal Medicine, College of Medicine, Chungbuk National University, Chungbuk 28644, Korea; kwon@chungbuk.ac.kr; 4 Department of Family Medicine, Yongin Severance Hospital, Yonsei University, Yongin 17046, Korea; bjpark96@yuhs.ac; 5 Department of Food Science and Biotechnology, College of Life Science, CHA University, Seongnam-si 13488, Korea

**Keywords:** ferritin, oxidative stress, inflammation, chronic kidney disease, iron

## Abstract

(1) Background: Oxidative stress and inflammation are associated with higher risk of chronic kidney disease (CKD). Serum ferritin concentrations correlate with total iron levels and systemic inflammation. (2) Methods: This study was cross-sectionally designed, based on the 2010–2012 Korean National Health and Nutrition Examination Survey (KNHANES). According to ferritin values, 13,462 participants (6082 men and 7380 women) were categorized into the normal- and high-ferritin groups (cut-off points: 200 ng/mL in men, 150 ng/mL in women). (3) Results: The mean ages of men and women were 44.5 and 48.4 years, respectively. The percentage of participants categorized into the high-ferritin group was 15.1% for men and 3.6% for women. The estimated glomerular filtration rate levels in the normal- and high-ferritin groups were 93.2 and 93.8 mL/min/1.73 m^2^ for men and 97.1 and 87.7 mL/min/1.73 m^2^ for women, respectively. The prevalence of CKD in the normal- and high-ferritin groups was 2.6% and 3.9% for men and 3.2% and 8.1% for women, respectively. Compared with the normal-ferritin group, the odds ratios (95% confidence intervals) for CKD of the high-ferritin group were 1.573 (1.014–2.441) in men and 1.061 (0.381–2.955) in women, after adjustments for age and other covariates. (4) Conclusions: High ferritin levels were associated with a higher risk of CKD in men but not in women.

## 1. Introduction

Chronic kidney disease (CKD) is becoming prevalent worldwide, affected by the global increase of the aged population. The burden of CKD on public health is increasing in severity [1]. In addition to aging, modifiable risk factors, such as obesity, cigarette-smoking, alcohol-drinking, and physical inactivity, are widely known causes of CKD. Furthermore, oxidative stress and inflammation interact with each other and can accelerate the development and progression of kidney injury [2].

The overloading of body iron plays a role as an oxidative stressor, which can convert less-reactive free radicals to more-reactive hydroxyl radicals. These active radicals can affect lipids, proteins, and deoxyribonucleic acid (DNA), resulting in tissue injury and dysfunction [3]. Excess iron causes oxidative stress and induces inflammation, leading to renal disease progression [4]. Serum ferritin levels correlate with total body iron storage and systemic inflammation [5]. The level of serum ferritin, an acute phase protein, is increased in an inflammatory environment [5]. Previous studies have reported that elevated serum ferritin levels are associated with insulin resistance syndrome, hypertension, dyslipidemia, obesity, and metabolic syndrome as risk factors of CKD [6,7,8,9,10]. Furthermore, elevated serum ferritin levels in hemodialysis patients predict higher mortality [11]. However, there is a lack of evidence whether serum ferritin levels are associated with risk of CKD.

The aim of this study is to investigate the associations between serum ferritin levels and CKD prevalence using the 2010–2012 Korean National Health and Nutrition Examination Survey (KNHANES).

## 2. Methods

### 2.1. Study Population

The Korean Ministry of Health and Welfare conducts the KNHANES, a nationwide survey representing the entire Korean population. The sampling units were chosen as households using a stratified, multistage, probability-sampling design according to geographic area, sex, and age group, based on household registries. Participants were requested to answer a health interview survey including a health behavior questionnaire, health examination questionnaire, and nutrition questionnaire. Trained interviewers conducted the health interview survey through one-on-one interviews at the participants’ houses. The National Health Enhancement Act guarantees the citizens’ right to refuse to participate in this survey. All procedures performed in studies involving human participants were in accordance with the ethical standards of the institutional and/or national research committee and with the 1964 Helsinki Declaration and its later amendments or comparable ethical standards. All participants in the survey were provided written informed consent. The KNHANES was approved by the Institutional Review Board of the KCDC (IRB No: 2008-04EXP-01-C, 2009-01CON-03-2C, 2010-02CON-21-C, 2011-02CON-06C), and written informed consent was collected from all of the participants before the KNHANES. The Korea Center for Disease Control and Prevention acquired participant approval for the further use of blood samples for academic research.

We excluded participants younger than 20 years, those who had incomplete laboratory data, and those whose ferritin level was lower than 15 ng/mL. After these exclusions, 13,462 individuals (6082 men and 7380 women) were included in the final analysis. The Institutional Review Board of the Korea Centers for Disease Control and Prevention approved this study.

### 2.2. Measurement of Anthropometry and Laboratory Data

Trained medical staff conducted physical examinations following standard protocols. Body weight and height were measured to the nearest 0.1 kg and 0.1 cm, respectively, with subjects wearing light indoor clothing without shoes. Blood pressure was measured twice on the right arm at five-minute intervals using a mercury sphygmomanometer (Baumanometer; Baum, Copiague, NY, USA) and was charted as a mean value. After participants fasted overnight, blood samples were collected from the antecubital vein. The levels of fasting plasma glucose, cholesterol, alanine aminotransferase (ALT), and creatinine were measured enzymatically using a Hitachi Automatic Analyzer 7600 (Hitachi, Tokyo, Japan). White blood cell (WBC) counts were assessed using laser flow cytometry methods by XE-2100 (Sysmex, Kobe, Japan). Serum ferritin levels were measured by immunoradiometric assay using a 1470 WIZARD gamma-counter (PerkinElmer, Kurku, Finland). Dipstick urinalyses were conducted with an Urisys 2400 (Roche, Mannheim, Germany).

### 2.3. Definition of Ferritin Levels, Chronic Kidney Disease and Health Behavior

Participants with hypoferritinemia (<15 ng/mL) were excluded in order to avoid including patients with anemia. The WHO indicated that ferritin levels over 200 ng/mL for men and 150 ng/mL for women are associated with a severe risk of iron overload [12]. Ferritin levels were categorized into two groups according to cut-off points (men: 200 ng/mL, women: 150 ng/mL): normal-ferritin group, 15–200 ng/mL in men and 15–150 ng/mL in women; and high-ferritin group, >200 ng/mL in men and >150 ng/mL in women [12]. An estimated glomerular filtration rate (eGFR) was calculated using the abbreviated equation from the Modification of Diet in Renal Disease (MDRD) study: eGFR (mL/min/1.73 m^2^) = 186.3 × (serum creatinine^−1.154^) × (age^−0.203^) × 0.742 (if female) [13]. CKD was defined based on either renal tissue damage or reduced renal function as eGFR < 60 mL/min/1.73 m^2^ or proteinuria 1+ or greater [13]. Proteinuria was tested using a dipstick method with freshly voided urine samples.

Information on health-related lifestyles was obtained from the data gathered from the self-report questionnaire during the interview portion of each survey. Individuals who engaged in ≥20 min of vigorous-intensity physical activity at least three days a week or ≥30 min of light- to moderate-intensity physical activity at least five days a week were categorized as the regular exercise group, using the short form of the International Physical Activity Questionnaire [14]. To identify high-risk drinkers, we used the Alcohol Use Disorders Identification Test (AUDIT) [15]. The cut-off points of the AUDIT score were used to categorize participants into three groups: low-risk drinkers, 0–7; intermediate-risk drinkers, 8–14; and high-risk drinkers, ≥15 points. Individuals who smoked cigarettes during the relevant survey period were categorized as current smokers. Daily calorie intake was monitored by a 24-h food recall and analyzed using CAN-Pro 3.0 software (Korean Nutrition Society, Seoul, Korea).

### 2.4. Statistical Analysis

Data taken from the Korea National Statistical Office was used to define the standard population. To represent the entire population of Korean adults with unbiased estimates, sampling weights were applied to account for the complex sampling. All data from continuous variables are presented as mean ± standard error (SE). Data from categorical variables are presented as percentage ± SE. Student’s *t*-tests for continuous variables and Chi-squared tests for categorical variables were used to compare the mean values and percentages according to ferritin level, respectively. The odds ratios (ORs) and corresponding 95% confidence intervals (95% CIs) for prevalence risk of CKD were calculated using weighted multivariate logistic regression analyses after adjusting for age and confounding factors. All analyses were conducted using SPSS statistical software, version 21 (IBM, New York, NY, USA). All statistical tests were two-tailed, and *p*-values less than 0.05 were considered statistically significant.

## 3. Results

Subject characteristics were presented in Table 1. The mean ages of men and women were 44.5 and 48.4 years old, respectively. The mean ferritin levels were 130.6 ng/mL in men and 57.7 ng/mL in women. The percentage of participants in the high-ferritin group was 15.1% for men and 3.6% for women. The eGFR levels of men and women were 93.2 and 96.8 mL/min/1.73 m^2^, respectively. The percentages of low eGFR and proteinuria were 2.0% and 1.2% in men and 2.3% and 1.2% in women, respectively. The prevalence of CKD was 2.8% in men and 3.4% in women.

Table 2 showed the subject characteristics according to ferritin levels. The high-ferritin group had higher levels of BMI, blood pressure, fasting plasma glucose, cholesterol, and ALT in both sexes. Female eGFR levels were lower in the high-ferritin group than the normal-ferritin group (97.1 vs. 87.7 mL/min/1.73 m^2^, *p*-value < 0.001), while male eGFR levels did not differ significantly between the groups (93.2 vs. 93.8 mL/min/1.73 m^2^, *p*-value = 0.404). The percentage of men having low eGFR levels was 2.0% in the normal-ferritin group and 2.2% in the high-ferritin group (*p*-value = 0.636), while that of women was 2.1% in the normal-ferritin group and 7.7% in the high-ferritin group (*p*-value < 0.001). The proteinuria percentage in men was 0.9% in the normal-ferritin group and 2.6% in the high-ferritin group (*p*-value < 0.001), while that of women were 1.2% in the normal-ferritin group and 0.9% in the high-ferritin group (*p*-value = 0.582). The percentage of individuals with CKD in both sexes was higher in the high-ferritin group than the normal-ferritin group (2.6% vs. 3.9% in men, *p*-value = 0.032; 3.2% vs. 8.1% in women, *p*-value < 0.001).

Weighted logistic regression analyses were conducted to demonstrate the association between ferritin levels and CKD risk, as shown in Table 3. Compared to the normal-ferritin group, OR (95% CI) of the high-ferritin group was 1.590 (1.035–2.444) in men and 1.438 (0.895–2.312) in women after being adjusted for age (Model 1). After adjustment for age, energy intake, drinking status, smoking status, physical activity status, BMI, fasting plasma glucose, cholesterol, and ALT, the OR (95% CI) was 1.573 (1.014–2.441) in men and 1.061 (0.381–2.955) in women (Model 3). After further adjusting for WBC counts as another inflammatory marker in addition to Model 3, the statistical significance of the OR (95% CI) for CKD in men was attenuated (1.524 (0.995–2.335), *p*-value = 0.053) in Model 4. Additionally, logistic regression analyses for CKD of various risk factors were conducted in Supplementary Materials Table S1.

## 4. Discussion

The major findings of this study are that serum ferritin levels were associated with a higher prevalence risk of CKD in Korean men but not in Korean women.

The prevalence of CKD is increasing, especially in Westernized countries. Conventionally, aging, elevated blood pressure, hyperglycemia, dyslipidemia, and obesity contribute to renal impairment. Unhealthy behaviors such as smoking tobacco, drinking alcohol, and a sedentary lifestyle are considered risk factors of CKD. CKD shares these risk factors with cardiovascular diseases and some cancers. Most patients with CKD die of those diseases; some of them progress to end-stage renal disease. Recent studies have reported that CKD was associated with oxidative stress and chronic inflammation [2]. In addition, oxidative stress and inflammation link CKD with atherosclerotic cardiovascular diseases [2]. Recently, the roles of inflammation and oxidative stress on CKD development and progression have been highlighted [16]. Oxidative stress, such as iron overload, can increase the inflammatory response, such as TNF-α and its receptors, adhesion molecules, and chemokine expression. The increased pro-inflammatory response interacts to stimulate oxidative stress. This interaction between oxidative stress and inflammation may have a synergistic effect on CKD development and progression. Ferritin can be considered a surrogate marker of oxidative stress and inflammation, but it cannot be considered to be their direct stimulator. In addition to iron overload as an indicator of oxidative stress, we used ferritin to represent the inflammatory status in CKD patients. This dual representativeness of ferritin can be a useful additional marker for the prediction of CKD.

Oxidative stress is a byproduct of aerobic respiration during mammalian energy production in the mitochondria. An oxidative environment in the body is also produced by iron overload and enzymatic or non-enzymatic sources such as nicotinamide dinucleotide (phosphate). Iron ions, directly or indirectly, interact with reactive oxygen species through oxidation-reduction and radical scavenging mechanisms. Iron in blood binds to transferrin for transport to various tissues. After transport, iron enters cells by means of the transferrin receptor, and excess iron is stored and regulated by ferritin [17]. Iron-related oxidative stress can catalyze the conversion of superoxide and hydrogen peroxide to more potent oxidants, such as hydroxyl radicals or ferryl or perferryl species, by Fenton-type reactions [18]. Additionally, iron activates NF-kB, resulting in TNF-α and macrophage inflammatory protein-1. Chronic iron overload in animal models decreases antioxidant activity, such as glutathione peroxidase, and increases lipid peroxidation, causing the oxidation of proteins and nucleic acids [19]. Young et al. reported that patients with hereditary hemochromatosis have reduced levels of antioxidant activity and increased lipid peroxidation [20]. Based on previous studies using animal and human models, iron overload can result in lipid, protein, and nucleic acid peroxidation and in reactive oxygen species in the circulation. Excessive iron causes endothelial dysfunction and inflammation. Oxidative stresses such as excessive iron are associated with diabetes mellitus, hypertension and dyslipidemia and may accelerate renal injury and dysfunction [2]. Ferritin as a surrogate marker of body iron levels, and inflammatory reactions can be considered as a pathophysiologic bridge among oxidative stress, inflammation, and CKD.

Chronic low-grade inflammation is identified as an integral part of the pathogenesis of CKD, although the precise mechanisms between inflammation and CKD have not yet been elucidated. Previous studies have shown that elevated white blood cell count and C-reactive protein level are associated with CKD and its progression [21,22]. Systemic inflammation causes the reduced production of nitric oxide (NO) and increased activity of the renin-angiotensin system, leading to endothelial dysfunction [2]. Reduced NO as an endogenous vasodilator contributes to the progression of renal damage. Furthermore, various pro-inflammatory cytokines exacerbate the progression of CKD.

Ferritin is the main iron storage protein in human and is classified into two types: L-ferritin, from the liver, promotes iron nucleation, mineralization and long-term storage; H-ferritin, from the heart, plays a role in detoxifying iron and is regulated by cytokines and hormones [23]. Body iron is primarily stored in the form of ferritin, which can surround and carry about 4000–4500 iron atoms. Serum ferritin concentrations are positively correlated with total body iron levels if acute inflammation is absent. Its measurement specifically represents body iron level. Exposure to excess iron results in expression of L-ferritin mRNA, while chelation of iron using defoxamine decreases L-ferritin mRNA level. The regulatory system of ferritin is sensitive to general oxidative status in addition to iron level [24]. As ferritin is an acute phase protein, its level is elevated under acute and chronic inflammatory environments, independent of iron status. The H-ferritin gene is expressed by pro-inflammatory cytokines such as tumor necrosis factor-α and interferon-γ [25]. Thus, ferritin is can be used as a surrogate marker and connecting link between iron storage and inflammatory status. High ferritin level implicates iron overload, resulting in oxidative stress, and also reflects inflammation. In addition, previous studies have reported that ferritin levels were associated with higher risk of cerebro-/cardio-vascular diseases, infection and mortality in hemodialysis patients [11,26].

The World Health Organization has suggested that the cut-off point of ferritin value by sex varies as 15 ng/mL for iron depletion in both sexes and 200 ng/mL in men and 150 ng/mL in women for iron excess [12]. To exclude subjects with anemia due to iron deficiency or chronic disease, we excluded participants with ferritin <15 ng/mL. While the cut-off ferritin level for men was higher than for women, the percentage of individuals in the high-ferritin group is higher in men than women (15.1% vs. 3.6%). In addition, men in the high-ferritin group exhibited unhealthier behaviors, such as high-risk drinking, cigarette-smoking, and physical inactivity, compared to the men in the normal-ferritin group, while the unhealthy behaviors of the women were not significantly different between the two groups. Unhealthier behaviors in men would have an additive effect on the higher risk of CKD prevalence.

Some limitations should be taken into account when interpreting this work. First, we may not conclude that higher ferritin level causes CKD based on this cross-sectional design; however, we can expect to draw the relationship between higher ferritin level and CKD by performing prospective cohort study. Interpretation of our results should consider its limitations with regard to drawing conclusions on any causal relationships between ferritin levels and CKD prevalence. Further longitudinal studies are warranted to better clarify this causal relationship. Second, the Kidney Disease Outcomes Quality Initiative guidelines [27] define CKD as the presence of either low eGFR or proteinuria lasting for at least three months. Because we used a single measurement of eGFR and proteinuria, we cannot confirm that they lasted for three month or more. Furthermore, the MDRD formula was used to define CKD as an eGFR less than 60 mL/1.73m^2^, rather than actually measuring the GFR. The MDRD formula was developed for people of European descents. Unfortunately, there is no equation to estimate GFR for Koreans. If a formula for the estimation of GFR in Korean population was available, we could more accurately analyze this association. Third, urine analyses were not based on the first morning urine samples. In addition, proteinuria was examined using dipstick methods rather than albuminuria measured by 24-h urine collection or albumin-to-creatinine ratio. Even though the Kidney Disease Improving Global Outcomes (KDIGO) guideline recommends the measurement of albuminuria for the evaluation of CKD, they allow the use of proteinuria measured by dipstick methods. Fourth, we could not classify ferritin into L-ferritin and H-ferritin. Thus, we do not know which form of ferritin is mainly associated with CKD prevalence. Fifth, an inadequate number of high-ferritin women and a low cut-off point for ferritin might have made it difficult to determine the effect of ferritin on CKD in women.

Despite these potential limitations, this study has several strengths. We preserved the representativeness of the Korean population because all analyses were conducted using data based on the 2010–2012 KNHANES after sampling weighting was applied. Ferritin level is easily measured in a clinical setting and is a surrogate marker reflective of oxidative stress and inflammation. Body ferritin level is rarely influenced by sea level or tobacco-smoking behavior.

## 5. Conclusions

In conclusion, serum ferritin concentrations seem to be associated with a higher risk of CKD in men but not in women, after adjustment for age, energy intake, drinking status, smoking status, physical activity status, BMI, fasting plasma glucose, cholesterol, and ALT. Ferritin concentration is one of the laboratory tests used in clinical settings to detect iron deficiency or overload. If a physician recognizes that his/her patients have high ferritin levels in the absence of acute inflammation, the doctor can recommend modification of risk factors, such as obesity, hypertension, dyslipidemia, and hyperglycemia, and eventually prevent CKD and its comorbidities.

## Figures and Tables

**Table 1:** Subject characteristics by sex.

Parameters	Men	Women
Unweighted N	6082	7380
Age (year)	44.5 ± 0.3	48.4 ± 0.3
BMI (kg/m^2^)	24.1 ± 0.58	23.6 ± 0.1
SBP (mmHg)	120.7 ± 0.3	117.1 ± 0.3
DBP (mmHg)	79.4 ± 0.2	74.2 ± 0.2
FPG (mg/dL)	98.6 ± 0.4	96.0 ± 0.3
Cholesterol (mg/dL)	188.2 ± 0.7	190.8 ± 0.6
ALT (IU/L)	27.2 ± 0.5	18.6 ± 0.3
Creatinine (mg/dL)	0.97 ± 0.003	0.72 ± 0.003
Ferritin (ng/mL)	130.6 ± 1.9	57.7 ± 0.8
Energy intake (kcal/day)	2461 ± 18	1686 ± 12
eGFR (mL/min/1.73 m^2^)	93.2 ± 0.3	96.8 ± 0.3
High-risk drinker (%) ^a^	25.5 ± 0.8	4.8 ± 0.4
Ever smoker (%) ^a^	76.2 ± 0.7	11.2 ± 0.5
Regular exerciser (%) ^a^	50.0 ± 0.9	43.8 ± 0.8
High ferritin group (%) ^a^	15.1 ± 0.6	3.6 ± 0.3
Low eGFR (%) ^a^	2.0 ± 0.2	2.3 ± 0.2
Proteinuria (%)	1.2 ± 0.1	1.2 ± 0.2
Chronic kidney disease (%)	2.8 ± 0.2	3.4 ± 0.3

All data are presented as mean or percentage ± standard error (SE) and their *p*-values are determined by independent *t*-tests and chi-square tests. ^a^ High-risk drinker: Alcohol Use Disorders Identification Test AUDIT ≥ 15; Ever smoker who had smoked at least 100 cigarettes; Regular exerciser: vigorous intensity ≥ 3 days and/or moderate intensity ≥ 5 days; High ferritin group: ferritin levels are ≥200 ng/mL for men, 150 ng/mL for women; Low eGFR < 60 mL/min/1.73 m^2^. Abbreviation: BMI, body mass index; SBP, systolic blood pressure; DBP, diastolic blood pressure; FPG, fasting plasma glucose; ALT, alanine aminotransferase; eGFR, estimated glomerular filtration rate.

**Table 2:** Subject characteristics according to ferritin levels.

Parameters	Men		*p*-Value	Women		*p*-Value
Ferritin levels	15–200 ng/mL	>200 ng/mL		15–150 ng/mL	>150 ng/mL	
Unweighted N	5157	925		7050	330	
Age (year)	44.3 ± 0.3	45.7 ± 0.6	0.026	47.9 ± 0.3	61.0 ± 0.9	<0.001
BMI (kg/m^2^)	23.9 ± 0.1	25.1 ± 0.1	<0.001	23.5 ± 0.1	25.1 ± 0.2	<0.001
SBP (mmHg)	120.2 ± 0.3	123.4 ± 0.6	<0.001	116.8 ± 0.3	126.7 ± 1.3	<0.001
DBP (mmHg)	79.0 ± 0.2	81.9 ± 0.5	<0.001	74.1 ± 0.2	76.3 ± 0.7	0.002
FPG (mg/dL)	97.5 ± 0.4	105.0 ± 1.0	<0.001	95.4 ± 0.3	112.1 ± 3.3	<0.001
Cholesterol (mg/dL)	187.1 ± 0.7	194.0 ± 1.7	<0.001	190.2 ± 0.6	204.9 ± 3.3	<0.001
ALT (IU/L)	24.1 ± 0.3	44.6 ± 2.7	<0.001	17.9 ± 0.2	36.6 ± 3.6	<0.001
Creatinine (mg/dL)	0.97 ± 0.003	0.96 ± 0.007	0.145	0.72 ± 0.003	0.78 ± 0.027	0.018
Ferritin (ng/mL)	97.2 ± 0.8	319.0 ± 8.0	<0.001	51.2 ± 0.5	230.4 ± 9.2	<0.001
Energy intake (kcal/day)	2452 ± 19	2517 ± 51	0.220	1692 ± 12	1520 ± 39	<0.001
eGFR (mL/min/1.73 m^2^)	93.2 ± 0.3	93.8 ± 0.7	0.404	97.1 ± 0.3	87.7 ± 1.4	<0.001
High-risk drinker (%) ^a^	22.6 ± 0.8	41.8 ± 2.1	<0.001	4.8 ± 0.4	3.9 ± 1.6	0.624
Ever smoker (%) ^a^	74.8 ± 0.8	84.1 ± 1.6	<0.001	11.2 ± 0.6	11.4 ± 2.4	0.934
Regular exerciser (%) ^a^	51.1 ± 0.9	43.6 ± 2.1	0.001	44.0 ± 0.8	36.8 ± 3.6	0.054
Low eGFR (%) ^a^	2.0 ± 0.2	2.2 ± 0.5	0.636	2.1 ± 0.2	7.7 ± 1.6	<0.001
Proteinuria (%)	0.9 ± 0.1	2.6 ± 0.6	<0.001	1.2 ± 0.2	0.9 ± 0.5	0.582
Chronic kidney disease (%)	2.6 ± 0.2	3.9 ± 0.7	0.032	3.2 ± 0.3	8.1 ± 1.7	<0.001

All data are presented as mean or percentage ± standard error (SE) and their *p*-values are determined by independent *t*-tests and chi-square tests. ^a^ High-risk drinker: AUDIT ≥ 15; Ever a smoker who had smoked at least 100 cigarettes; Regular exerciser: vigorous intensity ≥ 3 days and/or moderate intensity ≥ 5 days; Low eGFR < 60 mL/min/1.73 m^2^; Abbreviation: BMI, body mass index; SBP, systolic blood pressure; DBP, diastolic blood pressure; FPG, fasting plasma glucose; ALT, alanine aminotransferase; eGFR, estimated glomerular filtration rate.

**Table 3:** Odds ratios for chronic kidney disease according to ferritin levels.

Adjusted Models	Men		Women	
Ferritin levels	15–200 ng/mL	>200 ng/mL	15–150 ng/mL	>150 ng/mL
Model 1	1	1.590 (1.035–2.444)	1	1.438 (0.895–2.312)
Model 2	1	1.703 (1.092–2.656)	1	1.435 (0.751–2.741)
Model 3	1	1.573 (1.014–2.441)	1	1.061 (0.381–2.955)

Note: Model 1 was adjusted for age; Model 2 was adjusted for age, energy intake, drinking status, smoking status, and physical activity status; Model 3 was adjusted for age, energy intake, systolic blood pressure, body mass index, fasting plasma glucose, total cholesterol, ALT, drinking status, smoking status, and physical activity status.

## Supplementary Materials

The following are available online at www.mdpi.com/1660-4601/13/11/1058/s1, Table S1. Logistic regression analyses for CKD of various risk factors.

## Abbreviations

The following abbreviations are used in this manuscript:

CI	confidence interval
CKD	chronic kidney disease
DNA	deoxyribonucleic acid
KNHANES	Korean National Health and Nutrition Examination Survey
ALT	alanine aminotransferase
GFR	glomerular filtration rate
MDRD	Modification of Diet in Renal Disease
AUDIT	Alcohol Use Disorders Identification Test
