# Supplementary material for: Ferritin Level Is Positively Associated with Chronic Kidney Disease in Korean Men, Based on the 2010–2012 Korean National Health and Nutrition Examination Survey
Source: Int J Environ Res Public Health. 2016 Oct 29;13(11):1058. doi: 10.3390/ijerph13111058 (PMC5129268; doi:10.3390/ijerph13111058)
Supplement: Supplementary file 1 [file ijerph-13-01058-s001.pdf]

Supplementary Materials: Ferritin Level is Positively Associated with Chronic Kidney Disease in Korean Men, Based on the 2010–2012 Korean National Health and Nutrition Examination Survey

Hee-Taik Kang [1], John A Linton [2], Soon Kil Kwon [3], Byoung-Jin Park [4] and Jong Hun Lee [5,*]

Table S1. Logistic regression analyses for CKD of various risk factors.

Risk Factors	Men	Women
AUDIT ≥ 15 scores vs. AUDIT < 15 scores	0.896 (0.567–1.415)	1.185 (0.177–7.934)
Ever Smoker vs. Non-smoker	1.218 (0.705–2.103)	1.537 (0.892–2.647)
Regular exercise vs. Physical inactivity	0.853 (0.598–1.217)	0.812 (0.512–1.272)
Age, every 1 year	1.079 (1.065–1.094)	1.064 (1.040–1.088)
SBP, every 10 mmHg	1.109 (1.000–1.229)	1.010 (0.880–1.158)
BMI, every 1 kg/m^2	1.112 (1.041–1188)	0.995 (0.939–1.054)
Glucose, every 10 mg/dL	1.146 (1.096–1.198)	1.084 (1.012–1.160)
Total Cholesterol, 10 mg/dL	0.955 (0.902–1.012)	1.011 (0.942–1.086)
AST, every 10 IU/L	0.898 (0.750–1.076)	1.040 (0.837–1.292)
WBC counts, every 1000 cells/mL	1.173 (1.088–1.265)	1.245 (1.121–1.382)

Adjusted for age, energy intake, systolic blood pressure, body mass index, fasting plasma glucose, total cholesterol, ALT, drinking status, smoking status, and physical activity status, in addition to ferritin group. High-risk drinker: AUDIT ≥ 15. Ever smoker who had smoked at least 100 cigarettes. Regular exerciser: vigorous intensity ≥ 3days and/or moderate intensity ≥ 5 days.

© 2016 by the authors; licensee MDPI, Basel, Switzerland. This article is an open access article distributed under the terms and conditions of the Creative Commons by Attribution (CC-BY) license (http://creativecommons.org/licenses/by/4.0/).
